# ﻿Unveiling species diversity within early-diverging fungi from China I: three new species of *Backusella* (Backusellaceae, Mucoromycota)

**DOI:** 10.3897/mycokeys.109.126029

**Published:** 2024-10-14

**Authors:** Heng Zhao, Yong Nie, Bo Huang, Xiao-Yong Liu

**Affiliations:** 1 College of Life Sciences, Shandong Normal University, Jinan 250358, China Shandong Normal University Jinan China; 2 School of Ecology and Nature Conservation, Beijing Forestry University, Beijing 100081, China Beijing Forestry University Beijing China; 3 School of Civil Engineering and Architecture, Anhui University of Technology, Ma'anshan 243002, China Anhui University of Technology Ma'anshan China; 4 Anhui Provincial Key Laboratory for Microbial Pest Control, Anhui Agricultural University, Hefei 230036, China Anhui Agricultural University Hefei China; 5 State Key Laboratory of Mycology, Institute of Microbiology, Chinese Academy of Sciences, Beijing 100101, China Institute of Microbiology, Chinese Academy of Sciences Beijing China

**Keywords:** Fungal diversity, morphology, Mucorales, phylogeny, physiology

## Abstract

The species diversity of early-diverging fungi has long lagged behind that of higher fungi, posing a significant obstacle to our comprehensive understanding of the fungal kingdom. Our ongoing research endeavors aim to address this gap by exploring the species diversity of early-diverging fungi in China. In this study, we describe three novel species within the *Backusella*, namely *B.elliptica***sp. nov.**, *B.fujianensis***sp. nov.**, and *B.variispora***sp. nov.**, based on phylogenetic and morphological analyses. In the phylogenetic analysis of the ITS (internal transcribed spacer), LSU (large subunit of ribosomal RNA gene), and RPB1 (RNA polymerase II largest subunit gene) regions, the *B.elliptica* and *B.fujianensis* cluster closely with *B.gigacellularis*, *B.ovalispora*, and *B.solicola*, and the *B.variispora* is closely related to *B.locustae* and *B.pernambucensis*. Morphologically, *B.elliptica* is distinguished by elliptical sporangiospores, as well as cylindrical and hemispherical columellae. The *B.fujianensis* is characterized by elliptical sporangiospores, and various types of columellae such as hemispherical, subglobose, depressed globose and conical. The *B.variispora* is characterized by subglobose to globose sporangiospores, as well as hemispherical, subglobose to globose columellae. Additionally, the sporangiophores are long and monopodially branched in *B.elliptica* and *B.fujianensis*, while short and simple or sympodially branched in *B.variispora*. Physiologically, the maximum growth temperatures of *B.elliptica* (32 °C), *B.fujianensis* (35 °C), and *B.variispora* were (35 °C) were determined. With the inclusion of these newly described taxa, the total number of *Backusella* species known from China now stands at 12. Finally, we provide a key to facilitate the morphological identification of *Backusella* species from Asia.

## ﻿Introduction

Currently, there is a remarkable increase in the number of documented fungal species owing to advances in molecular evidence. For instance, the 10^th^ edition of the Dictionary of the Fungi in 2008 recorded approximately 100,000 species (Kirk et al. 2008), but now the Fungal Names database reported 156,781 species (assessed on March 7, 2024; Wang et al. 2023a).

Early-diverging fungi, also known as basal or lower fungi, are important in biotechnological areas, such as production of enzymes, lipids and antifungal proteins, and anaerobic members colonizing the digestive tracts of herbivorous vertebrates play a significant role in the breakdown of lignocellulosic feed (Flad et al. 2020). This group of fungi is well-known as pathogens for human, livestock and amphibians, causing diseases such as mucormycosis and chytridiomycosis (Voigt et al. 2021). They encompass a diverse array of evolutionary lineages, morphological characteristics, and ecological distributions, with 17 phyla currently recognized (Galindo et al. 2021; Voigt et al. 2021; Wijayawardene et al. 2022). However, compared to higher fungi (Ascomycota and Basidiomycota, 153,609 species; https://nmdc.cn/fungalnames/; assessed on March 7, 2024), there were significantly limited studies on the evolutionary relationship and species diversity of the early-diverging fungal lineages (Benny et al. 2016; Spatafora et al. 2016; Galindo et al. 2021; Voigt et al. 2021; Zhao et al. 2023a), with only 3,172 species documented (https://nmdc.cn/fungalnames/; assessed on March 7, 2024; Wang et al. 2023a).

In China, studies of early-diverging fungi mainly focused on Entomophthoromycota, Glomeromycota, Kickxellomycota, Mucoromycota, and Mortierellomycota. Notably, from 1980s to 2010s, R.Y. Zheng (Chinese Academy of Sciences), Z.Z. Li (Anhui Agricultural University), S.M. Ho (National Taipei University of Education) and their colleagues have been engaged in these groups of fungi for nearly half a century (Zheng and Chen 1986, 1998; Ho and Chen 1990; Ho 1995a, 1995b, 1996, 2000, 2001, 2002a, 2002b, 2003, 2004, 2006a, 2006b; Li et al. 1999; Li 2000; Liu et al. 2001, 2008; Ho and Chang 2003; Ho et al. 2004, 2007, 2008; Liu 2004; Ho and Hsu 2005; Ho and Benny 2007, 2008; Zheng et al. 2007; Ho and Kirk 2009; Ho and Chuang 2010; Wang et al. 2013, 2014; Zheng and Liu 2014; Liu and Zheng 2015), and in 2018, Zheng and Liu made a summary of 452 species of chytrid, zygomycotan, and glomeromycotan fungi in China (Zheng and Liu 2018). Since then, H. Zhao and his colleagues have contributed 109 new species and new records in Mucoromycota from China, including new species and new records of *Absidia* (Zhao et al. 2021, 2022a, 2022b, 2023a; Zong et al. 2021), *Backusella*, *Circinella* (Zhao et al. 2023a), *Cunninghamella* (Zhao et al. 2021, 2323a; Wang et al. 2022a), *Gongronella* (Wang et al. 2023b; Zhao et al. 2023a), *Lichtheimia*, *Mucor*, *Syncephalastrum* (Zhao et al. 2023a), and *Umbelopsis* (Wang et al. 2022b; Zhao et al. 2023a). During the same period, Y. Nie and his colleagues also described 15 new species and five new records of Entomophthoromycota from China (Nie et al. 2024), covering genera *Azygosporus* (Cai et al. 2021), *Capillidium* (Wang et al. 2010a; Nie et al. 2020a, 2022a), *Conidiobolus* s.s. (Wang et al. 2010a, b; Nie et al. 2017, 2020b, 2023), and *Neoconidiobolus* (Nie et al. 2012, 2016, 2018, 2021, 2022b). Up to now, early diverging fungi in China accommodated a total of 581 chytrid, zygomycotan, and glomeromycotan species. We expect to conduct a series of studies on the species diversity of early-diverging fungi, and this is the first article in the series, reporting new species of the genus *Backusella*.

*Backusella* was proposed by C. Hesseltine and J. Ellis in 1969, characterized by transitorily recurved sporangiophores, and classified within Backusellaceae, Mucorales, Mucoromycetes, and Mucoromycota (Ellis and Hesseltine 1969; Walther et al. 2013; Urquhart et al. 2021; Wijayawardene et al. 2022; Zhao et al. 2023a). Members of *Backusella* are widely distributed on various substrates, such as soil, litter, toads, wood, invertebrates, and herbivore dung (Santos et al. 2023; Zhao et al. 2023a). In the 20^th^ century, only three species were described in *Backusella*. From the beginning of this century, the species of *Backusella* rapidly increased with a total of 38 species being reported (www. indexfungorum.org; accessed on March 8, 2024; de Souza et al. 2014; Lima et al. 2016; Nguyen et al. 2021; Urquhart et al. 2021; de Lima et al. 2022; Hurdeal et al. 2022; Cordeiro et al. 2023; Santos et al. 2023; Zhao et al. 2023a). However, research in China was relatively limited, with only nine *Backusella* species reported, accounting for 23.68% (9/38; Zheng et al. 2013; Zhao et al. 2023a), and only 15 Chinese occurrences out of the worldwide 3,030 (less than 1%) in the Global Biodiversity Information Facility database (GBIF 2024). In this study, soil samples were collected from Fujian and Hainan Provinces, China, and subjected to the isolation and identification of early-diverging fungi. Subsequently, three novel species within the *Backusella* were delineated through comprehensive approaches involving morphology, molecular phylogeny, and maximum growth temperatures.

## ﻿Materials and methods

### ﻿Samples and strains

During the field trips in Fujian and Hainan Provinces, China, soil samples were collected for the isolation of early-diverging fungi strains. Fujian Province is located along the southeast coast of China and has the subtropical monsoon climate. The air temperature is significantly affected by the monsoon in Fujian Province, with warm winters and an average annual temperature ranging from 15.7 °C to 23.7 °C. The annual precipitation is relatively abundant in Fujian Province, generally between 1400 and 2000 millimeters, decreasing from southeast to northwest. Hainan Province is located at the southern of China, with a tropical monsoon maritime climate. The annual average temperature ranges from 22.5 °C to 25.6 °C, and the annual precipitation is 1500–2500 millimeters.

The isolation methods followed protocols as in previous studies (Zong et al. 2021; Zhao et al. 2022b, 2023a). In brief, 1 g soil was thoroughly suspended with 9 mL sterilized water. Subsequently, 100 μL of the soil suspension was incubated at 25 °C on plates containing potato dextrose agar (PDA: glucose 20 g/L, potato 200 g/L, agar 20 g/L, and pH 7) medium supplemented with antibiotics (streptomycin sulfate 100 mg/mL, and ampicillin 100 mg/mL). The plates were examined using a stereo microscope (SMZ1500, Nikon Corporation, Japan), and cultures exhibiting morphological characteristics were transferred to new plates containing PDA medium and the same antibiotics. Pure strains were obtained through three generations of subcultures. Finally, all living cultures (strains) were deposited at both Beijing Forestry University and Shandong Normal University, and dried cultures (specimens) were preserved in the Herbarium Mycologicum Academiae Sinicae, Beijing, China (HMAS).

### ﻿Morphology and maximum growth temperature

The pure cultures were incubated with PDA medium at 25 °C for seven days in darkness, followed by morphological observation and photography under a light microscope (ZEISS, Axioscope 5, Germany). The determination of maximum growth temperature was conducted using established methods (Zheng et al. 2007; Zong et al. 2021; Zhao et al. 2023a). Briefly, pure cultures were inoculated onto the center of the PDA plates and placed in a series of biochemical incubators with a temperature range of 25 °C to 45 °C in 5 °C increments. The cultures were observed every 12 hours. All strains were repeated three times. Once the approximate maximum growth temperature was determined, the temperature was gradually increased until the maximum growth temperature was accurate to within 1 °C.

### ﻿DNA extraction, PCR amplification, and sequencing

The internal transcribed spacers (ITS), large subunit (LSU) of nuclear ribosomal RNA gene, and largest subunit of RNA polymerase II (RPB1) were used for molecular identification. Firstly, the cultures were grown on PDA plates at 25 °C for one week, followed by extraction of total DNA from mycelia using the GO-GPLF-400 kit (GeneOnBio Corporation, Changchun, China), as per the manufacturer’s instructions. Secondly, the ITS, LSU, and RPB1 regions were amplified using the primer pairs ITS 5 (5′‐GGA AGT AAA AGT CGT AAC AAG G‐3′) and ITS 4 (5′‐TCC TCC GCT TAT TGATAT GC‐3′; White et al. 1990), LR0R (5′‐ACC CGC TGA ACT TAA GC‐ 3′) and LR7 (5′‐TAC TAC CAC CAA GAT CT‐3′; http://www.biology.duke.edu/fungi/mycolab/primers.htm), as well as Af (5′-GAR TGY CCD GGD CAY TTY GG-3′) and Cr (5′-CCN GCD ATN TCR TTR TCC ATR TA-3′), respectively. PCR protocols followed previous studies (Urquhart et al. 2021; Zhao et al. 2022a, 2023b). Thirdly, the PCR products were sequenced by the BGI Tech Solutions Beijing Liuhe Co., Limited (https://www.bgi.com/, Beijing, China). Finally, all sequences generated were checked using Geneious v.9.0.2 (Kearse et al. 2012).

### ﻿Phylogenetic analyses

ITS, LSU, and PRB1 sequences of *Backusella* and the outgroup *Absidiayunnanensis* were obtained from the GenBank database or sequenced in this work (Table 1). Each genetic locus was separately aligned using the MAFFT v.7 (Katoh and Standley 2013), and the poorly-aligned sites were trimmed. The ITS, LSU, and RPB1 regions were concatenated using PhyloSuit v.1.2.3 (Zhang et al. 2020) before phylogenetic analyses. The best optimal model of the concatenated dataset was estimated by ModelTest-NG v.0.1.7 (Darriba et al. 2020).

**Table 1.** Taxon information and GenBank accession numbers used in the phylogenetic analyses.

Species	Strains no.	Type	GenBank accession nos.			References
			ITS	LSU	rpb1	
*Backusellaaustraliensis*	UoMAU34	T	MK959062	MK958800	OP832444	Urquhart et al. (2021)
*B.azygospora*	URM 8065	T	MK625216	MK625222	OP832446	Crous et al. (2019)
*B.brasiliensis*	URM 8395	T	OM458082	OM458083	–	de Lima et al. (2022)
*B.chlamydospora*	CNUFC-HL7		MZ171386	MZ148710	OP832447	Nguyen et al. (2021)
*B.chlamydospora*	CNUFC-PS1	T	MZ171385	MZ148709	OP832448	Nguyen et al. (2021)
*B.circina*	CBS 128.70	T	JN206258	NG_058650	OP832449	Ellis and Hesseltine (1969)
*B.constricta*	URM 7322		KT937157	KT937156	OP832453	Lima et al. (2016)
*B.dichotoma*	CGMCC 3.16108	T	OL678137	**PP477411**	**PP709516**	Zhao et al. (2023a)
*B.dichotoma*	XY07504		OL678138	–	–	Zhao et al. (2023a)
*B.dispersa*	CBS 107.09	T	JN206269	MH866118	OP832454	Urquhart et al. (2021)
***B.elliptica***	**HZ86-1**	**T**	**PP477393**	**PP477403**	**PP709513**	**This study**
***B.elliptica***	**HZ86-2**		**PP477394**	**PP477404**	**PP709514**	**This study**
***B.fujianensis***	**HZ219-1**	**T**	**PP477391**	**PP477401**	**PP709511**	**This study**
***B.fujianensis***	**HZ219-2**		**PP477392**	**PP477402**	**PP709512**	**This study**
*B.gigacellularis*	CCIBt 3866	T	KF742415	KF742414	–	de Souza et al. (2014)
*B.gigaspora*	CBS 538.80	T	HM999964	HM849692	OP832458	Cordeiro et al. (2023)
*B. “groupX*”	UoMAU121		MK959103	MK958792	OP832460	Urquhart et al. (2021)
*B. “groupX*”	UoMAU152		MK959102	MK958791	OP832461	Urquhart et al. (2021)
*B.grandis*	CBS 186.87	T	JN206252	JN206527	OP832496	Walther et al. (2013)
*B.indica*	CBS 786.70		JN206255	MH871743	OP832464	Walther et al. (2013)
*B.koreana*	CNUFC-CM05	T	MZ171387	MZ148711	OP832465	Nguyen et al. (2021)
*B.koreana*	CNUFC-CM06		MZ171388	MZ148712	OP832466	Nguyen et al. (2021)
*B.lamprospora*	CBS 118.08	T	NR_145291	NG_058650	OP832467	(Benny and Benjamin 1975)
*B.liffmaniae*	UoMAU58	T	MK959065	MK958734	OP832468	Urquhart et al. (2021)
*B.locustae*	EML-SFB2	T	KY449291	KY449292	OP832471	Wanasinghe et al. (2018)
*B.luteola*	UoMAU6	T	MK959058	MK958795	OP832472	Urquhart et al. (2021)
*B.macrospora*	UoMAU7	T	MK959107	MK958628	OP832474	Urquhart et al. (2021)
*B.mclennaniae*	UoMAU11		MK959077	MK958776	OP832476	Urquhart et al. (2021)
*B.mclennaniae*	UoMAU12	T	MK959078	MK958777	–	Urquhart et al. (2021)
*B.moniliformis*	CGMCC 3.16109	T	OL678139	**PP477412**	**PP709517**	Zhao et al. (2023a)
*B.morwellensis*	UoMAU16	T	MK959059	MK958808	OP832479	Urquhart et al. (2021)
*B.obliqua*	URM 8427	T	ON858475	ON858467	–	de Lima et al. (2022)
*B.oblongielliptica*	CBS 568.70	T	NG_076761	MH871630	OP832480	Walther et al. (2013)
*B.oblongielliptica*	XY08767		OL620091	–	–	Zhao et al. (2023a)
*B.oblongielliptica*	XY08768		OL620092	–	–	Zhao et al. (2023a)
*B.oblongispora*	CBS 569.70	T	JN206251	JN206407	OP832481	Walther et al. (2013)
*B.ovalispora*	CGMCC 3.16110	T	OL678140	–	–	Zhao et al. (2023a)
*B.ovalispora*	XY07481		OL678141	–	–	Zhao et al. (2023a)
*B.paraconstricta*	URM 8637	T	OQ625517	OQ625516	–	Santos et al. (2023)
*B.parvicylindrica*	UoMAU35	T	MK959109	MK958727	OP832482	Urquhart et al. (2021)
*B.pernambucensis*	URM 7647	T	OP339860	OP339863	OP832483	Cordeiro et al. (2023)
*B.pernambucensis*	URM 7648		OP339861	OP339864	OP832484	Cordeiro et al. (2023)
*B.psychrophila*	UoMAU55	T	MK959093	MK958749	–	Urquhart et al. (2021)
*B.recurva*	CBS 196.71		JN206265	JN206523	–	Urquhart et al. (2021)
*B.recurva*	CBS 318.52	ET	JN206261	JN206522	OP832488	Urquhart et al. (2021)
*B.solicola*	MFLUCC 22-0067	T	ON899832	ON892503	–	Hurdeal et al. (2022)
*B.tarrabulga*	UoMAU5	T	MK959060	MK958804	OP832490	Urquhart et al. (2021)
*B.thermophila*	CNUFC-CS02	T	MZ171389	MZ148713	OP832492	Nguyen et al. (2021)
*B.thermophila*	CNUFC-CS03		MZ171390	MZ148714	OP832493	Nguyen et al. (2021)
*B.tuberculispora*	CBS 562.66	LT	JN206267	JN206525	OP832494	Walther et al. (2013)
*B.tuberculispora*	CBS 570.70		JN206266	MH871631	OP832495	Walther et al. (2013)
*B.variabilis*	CBS 564.66	LT	JN206254	JN206528	OP832497	Walther et al. (2013)
***B.variispora***	**HZ69**	**T**	**PP477395**	**PP477405**	**PP709515**	**This study**
***B.variispora***	**HZ105**		**PP477396**	**PP477406**	–	**This study**
***B.variispora***	**HZ141**		**PP477397**	**PP477407**	–	**This study**
***B.variispora***	**HZ195**		**PP477398**	**PP477408**	–	**This study**
***B.variispora***	**HZ286**		**PP477399**	**PP477409**	–	**This study**
***B.variispora***	**HZ365**		**PP477400**	**PP477410**	–	**This study**
*B.westeae*	UoMAU4	T	MK959061	MK958796	OP832498	Urquhart et al. (2021)
*A.yunnanensis*	CGMCC 3.16259	T	ON074700	ON074687	–	Zhao et al. (2022)
*A.yunnanensis*	XY09528		ON074701	ON074688	–	Zhao et al. (2022)

Note: “T”, ‘ET”, and “LT” are represented ex-type, ex-epitype, and ex-lectotype, respectively. “–” is represented absences of sequences.

Maximum Likelihood (ML) and Bayesian Inference (BI) phylogenetic analyses were conducted with RAxML v.8 (Stamatakis 2014) and MrBayes v.3.2.7a (Ronquist et al. 2012), respectively, following the methods described in previous studies (Nie et al. 2020a, 2020b; Zhao et al. 2023a). For ML analysis, 1,000 bootstrap replications were conducted using the best optimal model. For BI analysis, two million generations were run until the standard deviation fell below 0.01, and the first 25% were discarded as burn-in. Meanwhile, ML and BI analyses were carried out using ITS and LSU sequences. Finally, the ML and BI trees were visualized using the Figtree v1.4.4 (http://tree.bio.ed.ac.uk/software/figtree/). Nodes with ML bootstrap values below 50% and BI posterior probability values of less than 0.9 were not considered.

## ﻿Results

### ﻿Phylogeny

The concatenated dataset comprised a total of 2,685 characters derived from 61 strains, including 1,029 characters from ITS sequences, 661 characters from LSU sequences, and 995 characters from RPB1 sequences (Suppl. material 1). A concatenated dataset of ITS and LSU sequences was provided in the supplementary material Suppl. material 2. GTR+I+G model was selected as the most suitable for the analysis. For the BI analysis, the standard deviation was 0.004813 after two million generations were calculated.

**Figure 1.**
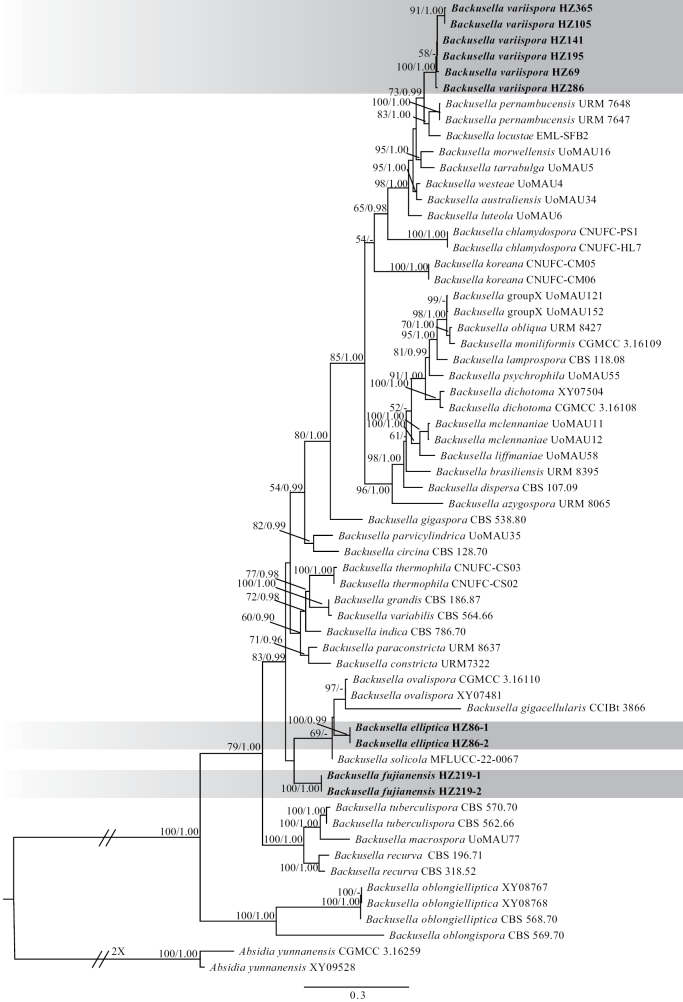
The Maximum Likelihood phylogenetic tree of the genus *Backusella* based on ITS, LSU, and RPB1 genetic loci. Two strains of *Absidiayunnanensis* serve as the outgroups. The new species, *Backusellafujianensis*, *B.elliptica*, and *B.variispora*, are shaded. The Maximum Likelihood bootstrap values (MLBV ≥ 50%) / Bayesian Posterior Probabilities (BPP ≥ 0.90) of each clade are indicated along branches. Some branches are shortened to fit to the page, which are indicated by double slashes and the number of fold times. The scale bar at the bottom left indicates the number of substitutions per site.

Phylogenetic analyses of the *Backusella* suggested that three new species, namely *B.elliptica*, *B.fujianensis*, and *B.variispora*, were well supported (Fig. 1, Suppl. material 3). The *B.elliptica* and *B.fujianensis* formed a distinct clade with *B.gigacellularis*, *B.ovalispora*, and *B.solicola*. The *B.variispora* was sister to *B.locustae* and *B.pernambucensis* (MLBV 73% / BPP 0.99).

### ﻿Taxonomy

#### ﻿ Backusella elliptica

Taxon classificationFungiMucoralesBackusellaceae

H. Zhao & X.Y. Liu sp. nov.

CD916D96-7E33-5503-929B-5ACE0755E359

Fungal Names: FN 571901

Fig. 2

##### Etymology.

*elliptica* (Lat.) refers to the species having elliptical sporangiospores.

##### Holotype.

HMAS 352890.

Colonies on PDA at 25 °C for 4 days, reaching 90 mm in diameter, more than 15 mm high, flat, granulate, initially white, soon becoming pale mouse-grey, reverse straw-yellow stramineus. Hyphae aseptate at first, septate with age, hyaline, 5.0–18.5 μm in diameter. Rhizoids absent. Stolons absent. Long sporangiophores arising directly from substrate mycelia or aerial mycelia, transitorily curved, monopodially branched, usually with large terminal sporangia, erect, bent or rarely curved. Sporangia globose, hyaline to brownish, rough-walled, multi-spored, with more than 50 sporangiospores per sporangium, deliquescent-walled, 75.0–95.0 μm in diameter. Short sporangiophores unbranched, curved, ending with a multi-spored sproangiolum. Multi-spored sporangiola globose, hyaline, containing more than 10 sporangiospores, 30.0–50.0 μm in diameter, persistent-walled. Uni-spored sporangiola unknown. Apophyses rarely present. Collars, if present, small. Columellae usually cylindrical and rarely hemispherical, hyaline, with small droplets, 27.0–54.5 × 20.0–43.5 μm on the top of long sporangiophores, and usually conical, hyaline, with small droplets, 20.0–30.0 × 10.0–20.0 μm on the short sporangiophores. Sporangiospores elliptical, hyaline, with small droplets, 11.0–16.5 × 6.5–8.5 μm wide. Azygosporangia absent. Chlamydospores absent. Zygospores absent.

**Figure 2.**
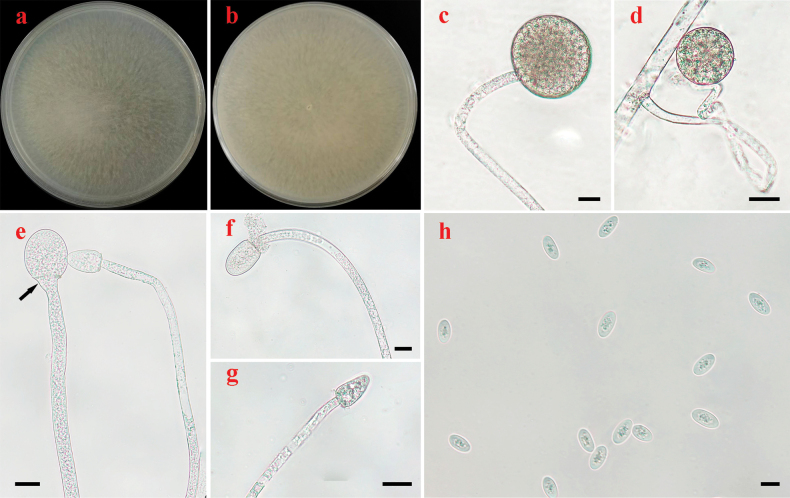
Morphologies of *Backusellaelliptica* ex-holotype HZ86-1 **a, b** colonies on PDA (**a** obverse **b** reverse) **c** long sporangiophores with multi-spored sporangia **d** short sporangiophores with multi-spored sporangia **e–g** sporangiophores with columellae **h** sporangiospores. Scale bars: 20 μm (**c–g**); 10 μm (**h**).

##### Materials examined.

China • Hainan Province, Ledong Li Autonomous Country, 18°42'35"N, 108°52'36"E, from forest soil sample, 11 April 2023, Heng Zhao (holotype HMAS 352890, living ex-holotype culture HZ86-1, and living culture HZ86-2).

##### GenBank accession numbers.

ITS, PP477393 and PP477394; LSU, PP477403 and PP477404, RPB1, PP709513 and PP709514.

##### Maximum growth temperature.

32 °C.

#### ﻿ Backusella fujianensis

Taxon classificationFungiMucoralesBackusellaceae

H. Zhao & X.Y. Liu sp. nov.

0A30502B-18E4-5F5B-B2CC-EDE3C0761A86

Fungal Names: FN 571900

Fig. 3

##### Etymology.

fujianensis (Lat.) refers to Fujian province where the type was collected.

##### Holotype.

HMAS 352889.

Colonies on PDA at 25 °C for 4 days, reaching 90 mm in diameter, more than 15 mm high, granulate, lobed and scaly, initially white, soon becoming pale mouse-grey, reverse straw-yellow stramineus. Hyphae aseptate at first, septate with age, hyaline, 5.5–25.5 μm in diameter. Rhizoids absent. Stolons absent. Long sporangiophores arising directly from substrate or aerial mycelia, transitorily curved, monopodially branched, usually with large terminal sporangia, erect, bent or curved. Sporangia subglobose to globose, hyaline to brownish, rough-walled, multi-spored, with more than 50 sporangiospores per sporangium, persistent-walled, 70.0–160.0 μm in diameter. Short sporangiophores unbranched, ending with a multi-spored sporangiolum. Multi-spored sporangiola subglobose to globose, hyaline, containing more than 20 sporangiospores, 45.0–65.0 μm in diameter, persistent-walled. Uni-sporangiola unknown. Apophyses absent. Collars if present, small. Columellae hemispherical, depressed globose to subglobose, hyaline to light brown, 36.0–64.5 × 33.0–63.5 μm in long sporangiophores, and conical and hemispherical, hyaline, 13.0–21.0 × 12.0–20.0 μm in short sporangiophores. Sporangiospores elliptical, rarely irregular, hyaline, with droplets, 12.0–21.5 × 6.0–10.5 μm. Azygosporangia absent. Chlamydospores absent. Zygospores absent.

**Figure 3.**
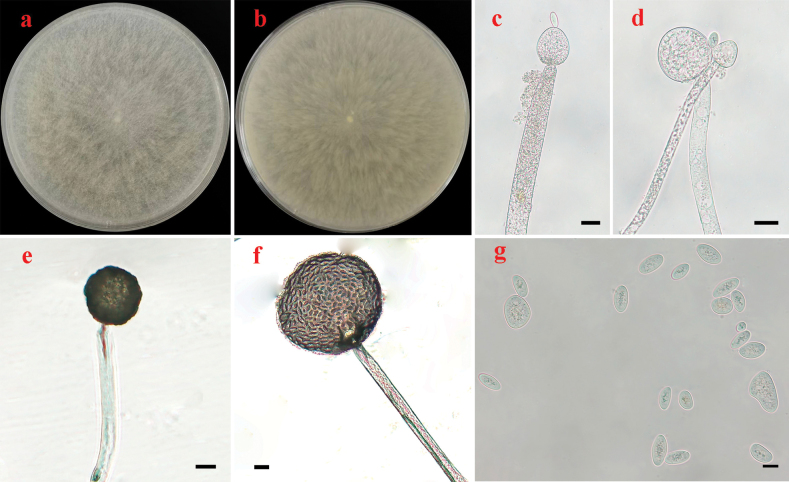
Morphologies of *Backusellafujianensis* ex-holotype HZ219-1 **a, b** colonies on PDA (**a** obverse **b** reverse) **c, d** sporangiophores with columellae **e** short sporophore with multi-spored sporangia **f** tall sporophore with multi-spored sporangia **g** sporangiospores. Scale bars: 20 μm (**c–f**); 10 μm (**g**).

##### Materials examined.

China • Fujian Province, Wuyishan City, 27°48'59"N, 117°42'46"E, from forest soil sample, 15 October 2022, Heng Zhao (holotype HMAS 352889, living ex-holotype culture HZ219-1, and living culture HZ219-2).

##### GenBank accession numbers.

ITS, PP477391 and PP477392; LSU, PP477401 and PP477402, RPB1, PP709511 and PP709512.

##### Maximum growth temperature.

35 °C.

#### ﻿ Backusella variispora

Taxon classificationFungiMucoralesBackusellaceae

H. Zhao & X.Y. Liu sp. nov.

4A18494A-04C8-579F-A256-B08D2353EC95

Fungal Names: FN 571902

Fig. 4

##### Etymology.

*variispora* (Lat.) refers to the species having an uneven size of sporangiospores.

##### Holotype.

HMAS 352891.

Colonies on PDA at 25 °C for 4 days, reaching 90 mm in diameter, more than 15 mm high, flat, granulate, initially white, soon becoming pale mouse-grey, irregular at margin. Hyphae aseptate at first, septate with age, hyaline, 4.5–11.5 μm in diameter. Rhizoids absent. Stolons absent. Long sporangiophores arising directly from substrate mycelia, transitorily curved, monopodially branched, with large terminal sporangia, erect, bent or curved. Sporangia globose, hyaline to brownish, wall rough with spines, deliquescent, rough, multi-spored, with more than 20 sporangiospores per sporangium, 30.5–60.0 μm in diameter. Short sporangiophores simple or sympodial, ending with a multi-spored. Multi-spored sporangiola subglobose to globose, with numerous spines, hyaline, containing 5–10 sporangiospores, persistent-walled, 14.5–26.0 μm in diameter. Apophyses absent. Collars absent. Columellae hemispherical, subglobose to globose, hyaline, 21.0–32.5 × 20.0–33.0 μm in long sporangiophores, conical and hemispherical, hyaline, 14.5–18.5 × 14.0–18.0 μm in short sporangiophores. Sporangiospores subglobose to globose, hyaline, with droplets, 5.0–16.0 μm in diameter. Azygosporangia absent. Chlamydospores absent. Zygospores absent.

**Figure 4.**
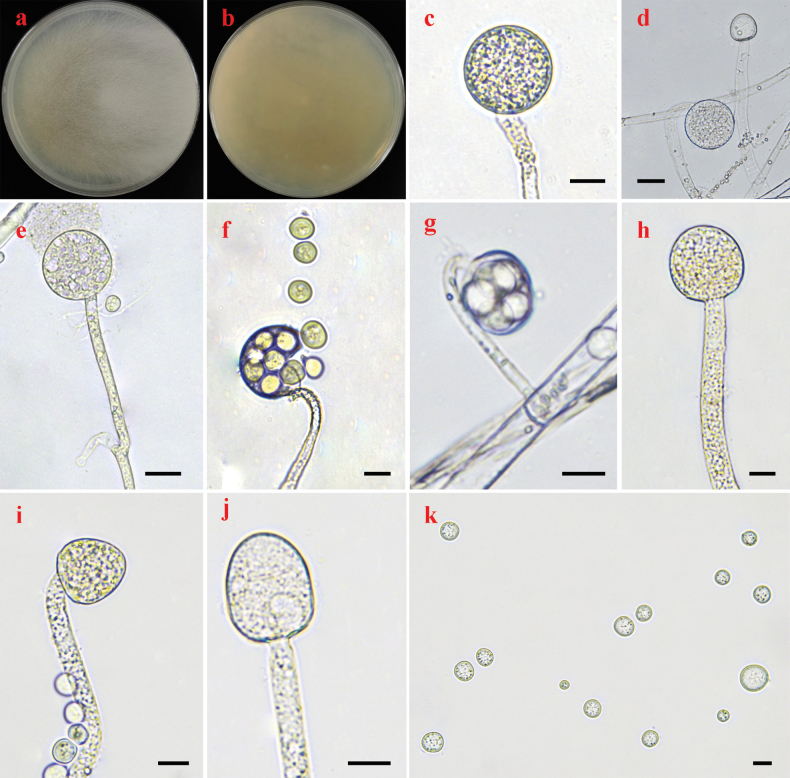
Morphologies of *Backusellavariispora* ex-holotype HZ69 **a, b** colonies on PDA (**a** obverse **b** reverse) **c–e** long sporangiophores with multi-spored sporangia **f, g** short sporangiophores with multi-spored sporangia **h–j** sporophore with columellae **k** sporangiospores. Scale bars: 10 μm (**c, f–k**); 20 μm (**d, e**).

##### Materials examined.

China • Hainan Province, Ledong Li Autonomous County, 18°42'35"N, 108°52'36"E, from soil sample, 11 April 2023, (holotype HMAS 352891, living ex-holotype culture HZ69) • Changjiang Li Autonomous County, 19°7'18"N, 109°7'7"E, from soil sample, 12 April 2023, Heng Zhao (living cultures HZ105, HZ195, and HZ365) • Lingshui Li Autonomous County, 18°42'8"N, 109°50'13"E, from forest soil sample, 9 April 2023, Heng Zhao (living cultures HZ141 and HZ286).

##### GenBank accession numbers.

ITS, PP477395–PP477400; LSU, PP477405–PP477410, RPB1, PP709511 and PP709515.

##### Maximum growth temperature.

35 °C.

## ﻿Discussion

In this study, three novel species, *Backusellafujianensis*, *B.elliptica*, and *B.variispora* were proposed based on phylogenetic relationships, morphological characteristics, and maximum growth temperatures. Phylogenetic analyses showed that the *B.elliptica* and *B.fujianensis* are closely related to *B.gigacellularis*, *B.ovalispora*, and *B.solicola*, and the *B.variispora* is closely related to *B.locustae* and *B.pernambucensis*.

These three new species are morphologically distinguished from their closely-related species. In detail, the *B.gigacellularis* differs from *B.elliptica* by fewer sporangiospores in multi-spored sporangiola (3–4 vs. more than 10), the absence of collars, the presence of giant cells, and the irregular sporangiospores (de Souza et al. 2014). The *B.ovalispora* differs from *B.fujianensis* by a faster growth speed (3d vs. 4d reaching 90 mm on PDA), the presence of uni-spored sporangiola, less sporangiospores in multi-spored sporangiola (3–4 vs. more than 10), and globose or subglobose columellae (Zhao et al. 2023a). The *B.solicola* differs from *B.elliptica* by subglobose columellae, fewer sporangiospores of multi-spored sporangiola (4–8 vs. more than 10), and the presence of the uni-spored sporangiola, chlamydospores, and rhizoids (Hurdeal et al. 2022).

The *B.gigacellularis* differs from *B.fujianensis* by the fewer multi-spored sporangiola (up to 23 μm in diameter vs. 43–64 μm in diameter) and fewer sporangiospores (3–4 vs. more than 20), the absence of collar, and the presence of giant cells (de Souza et al. 2014). The *B.ovalispora* differs from *B.fujianensis* by faster growth speed (3d vs. 4d reaching 90 mm on PDA), the presence of uni-spored sporangiola, less sporangiospores in multi-spored sporangiola (3–4 vs. more than 20; Zhao et al. 2023a). The *B.solicola* differs from *B.fujianensis* by forming oblong to cylindrical columellae, fewer sporangiospores in multi-spored sporangiola (4–8 vs. more than 20), and the presence of the unispored sporangiola, chlamydospores, and rhizoids (Hurdeal et al. 2022). In addition, the *B.elliptica* differs from *B.fujianensis* by the absence of depressed globose to subglobose columellae, the presence of apophyses, and the lower maximum growth temperature (32 °C vs. 35 °C). The *B.locustae* differs from *B.variispora* by the larger sporangiospores (9–23.5 × 10.5–25.5 μm vs. 14.5–26.0 μm in diameter) and multi-spored sporangiola (31–59 × 33.5–61.5 μm vs. 5.0–16.0 μm in diameter; Wanasinghe et al. 2018). The *B.pernambucensis* differs from *B.variispora* by the presence of rhizoids and giant cells, and more sporangiospores in multi-spored sporangiola (up to 15 vs. 5–10; Cordeiro et al. 2023).

Recent studies have highlighted the significance of maximum growth temperature as a distinguishing characteristic among *Backusella* species. These studies have categorized maximum growth temperatures into three groups: no higher than 33 °C; between 33 °C and 35 °C; 36 °C or higher (Cordeiro et al. 2023; Santos et al. 2023). In this study, maximum growth temperatures of *B.fujianensis*, *B.elliptica*, and *B.variispora* were 35 °C, 32 °C, and 35 °C, respectively. However, it’s worth noting that the grouping based on maximum growth temperature is not entirely consistent with the results of the phylogenetic analyses (Cordeiro et al. 2023).

*Backusella* species are distributed around the world, such as in Brazil (13 species; Cordeiro et al. 2023; Santos et al. 2023), Australia (10 species; Urquhart et al. 2021), South Korea (seven species; Wanasinghe et al. 2018; Nguyen et al. 2021), and Thailand (one species; Hurdeal et al. 2022). Although the study of *Backusella* species diversity was carried out relatively late in China (Zheng et al. 2013; Zhao et al. 2023a), 12 species have been discovered, including the three novel species in this study. A total of 21 *Backusella* species were reported from Asia (Walther et al. 2013; Zheng et al. 2013; Wanasinghe et al. 2018; Nguyen et al. 2021; Hurdeal et al. 2022; Zhao et al. 2023a). Since the characters of *Backusellagranulispora* were unavailable, we provide herein a synoptic key to the other 20 Asian *Backusella* species.

### ﻿Key to species of *Backusella* from Asia

**Table:** 

1	Sporangiospores mainly subglobose to globose, ovoid, or irregularly polyhedral	**2**
–	Sporangiospores mainly ellipsoidal	**11**
2	Sporangiospores mainly ovoid or irregularly polyhedral	**3**
–	Sporangiospores mainly subglobose to globose	**4**
3	Sporangiospores mainly ovoid	***B.ovalispora***
–	Sporangiospores mainly irregularly polyhedral	***B.tuberculispora***
4	Azygosporangia subglobose to globose	***B.dichotoma***
–	Azygosporangia absent	**5**
5	Chlamydospores abundant in substrate hyphae, in chains	**6**
–	Chlamydospores absent	**7**
6	Short sporangiophores simple or rebranched; uni-spored 13.5–23.0 μm; columellae variable in shape, including subglobose, conical, ellipsoidal, cylindrical, hemispherical, near pyriform, or sometimes bell-shaped, long conical	***B.chlamydospora***
–	Short sporangiophores simple or simple or sympodial; uni-spored 23.5–40.0 μm; columellae hemispherical or conical	***B.moniliformis***
7	Uni-spored present, subglobose to globose	**8**
–	Uni-spored absent	**10**
8	Giant cells present, globose to oval	***B.koreana***
–	Giant cells absent	**9**
9	Uni-spored sporangiola are quite common, 18−24 μm in diameter; multi-spored sporangiola 13−33 μm in diameter	***B.circina***
–	Uni-spored sporangiola are rare, 9−14 μm in diameter; multi-spored sporangiola 14−41 μm in diameter	***B.lamprospora***
10	Multi-spored sporangiola contain roughly 4–25 sporangiospores, 31.0–59.0 × 33.5–61.5 μm	***B.locustae***
–	Multi-spored sporangiola contain roughly 5–10 sporangiospores, 14.5–26.0 μm in diameter	***B.variispora***
11	Chlamydospores abundant	***B.solicola***
–	Chlamydospores absent	**12**
12	Giant cells present	**13**
–	Giant cells absent	**15**
13	Presence of cylindrical columellae, 62 × 58 µm	***B.indica***
–	Absences of cylindrical columellae	**14**
14	Sporangiospores globose to broadly ellipsoid, 8–12 × 7–10 µm	***B.dispersa***
–	Sporangiospores oblongly ellipsoidal, in young cultures rather uniform, 39.2–40.5 × 14.9–15.5 µm, in ageing cultures smaller spores, 14 × 5 µm and up	***B.oblongielliptica***
15	Uni-spored rare, globose, up to 15 μm diameter	***B.thermophila***
–	Uni-spored absent	**16**
16	Columellae no more than 70 µm	**17**
–	Columellae up to 70 µm	**18**
17	Columellae depressed globose to subglobose, apophysate, maximum growth temperature 35 °C	***B.fujianensis***
–	Columellae usually cylindrical, nonapophysate, maximum growth temperature 32 °C	***B.elliptica***
18	Presence of pyriform columellae, up to 110 × 75 µm	***B.oblongispora***
–	Absences of pyriform columellae	**19**
19	Sporangia up to 250(-300) µm in diameter, columella conical to cylindrical-ellipsoidal, 115–200 × 100–180 µm	***B.grandis***
–	Sporangia up to 100(-150) µm in diameter, columella applanate conical or cylindrical, 70 × 75 (85 × 100) µm	***B.variabilis***

## Supplementary Material

XML Treatment for Backusella elliptica

XML Treatment for Backusella fujianensis

XML Treatment for Backusella variispora

## ﻿Additional information

### Author ORCIDs

Heng Zhao https://orcid.org/0000-0003-2938-5613

Yong Nie https://orcid.org/0000-0001-8964-1661

Bo Huang https://orcid.org/0000-0001-6032-7396

Xiao-Yong Liu https://orcid.org/0000-0002-8808-010X

### Data availability

The sequences were deposited in the GenBank database (Table 1).

## Supplementary materials

Supplementary material 1 The concatenated sequences of ITS, LSU, and RPB1 regions Data type phy https://binary.pensoft.net/file/1153581This dataset is made available under the Open Database License (http://opendatacommons.org/licenses/odbl/1.0/). The Open Database License (ODbL) is a license agreement intended to allow users to freely share, modify, and use this Dataset while maintaining this same freedom for others, provided that the original source and author(s) are credited.Heng Zhao, Yong Nie, Bo Huang, Xiao-Yong Liu

Supplementary material 2 The concatenated sequences of ITS and LSU regions https://doi.org/10.3897/mycokeys.109.126029.suppl2 Data type phy https://binary.pensoft.net/file/1153582This dataset is made available under the Open Database License (http://opendatacommons.org/licenses/odbl/1.0/). The Open Database License (ODbL) is a license agreement intended to allow users to freely share, modify, and use this Dataset while maintaining this same freedom for others, provided that the original source and author(s) are credited.Heng Zhao, Yong Nie, Bo Huang, Xiao-Yong Liu

Supplementary material 3 The Maximum Likelihood phylogenetic tree of the genus *Backusella* based on ITS and LSU genetic loci https://doi.org/10.3897/mycokeys.109.126029.suppl3 Data type pdf https://binary.pensoft.net/file/1153583This dataset is made available under the Open Database License (http://opendatacommons.org/licenses/odbl/1.0/). The Open Database License (ODbL) is a license agreement intended to allow users to freely share, modify, and use this Dataset while maintaining this same freedom for others, provided that the original source and author(s) are credited.Heng Zhao, Yong Nie, Bo Huang, Xiao-Yong Liu
