# Supplementary figures and images for: ﻿Unveiling species diversity within early-diverging fungi from China I: three new species of Backusella (Backusellaceae, Mucoromycota)
Source: MycoKeys. 2024 Oct 14;109:285–304. doi: 10.3897/mycokeys.109.126029 (PMC11494212; doi:10.3897/mycokeys.109.126029)

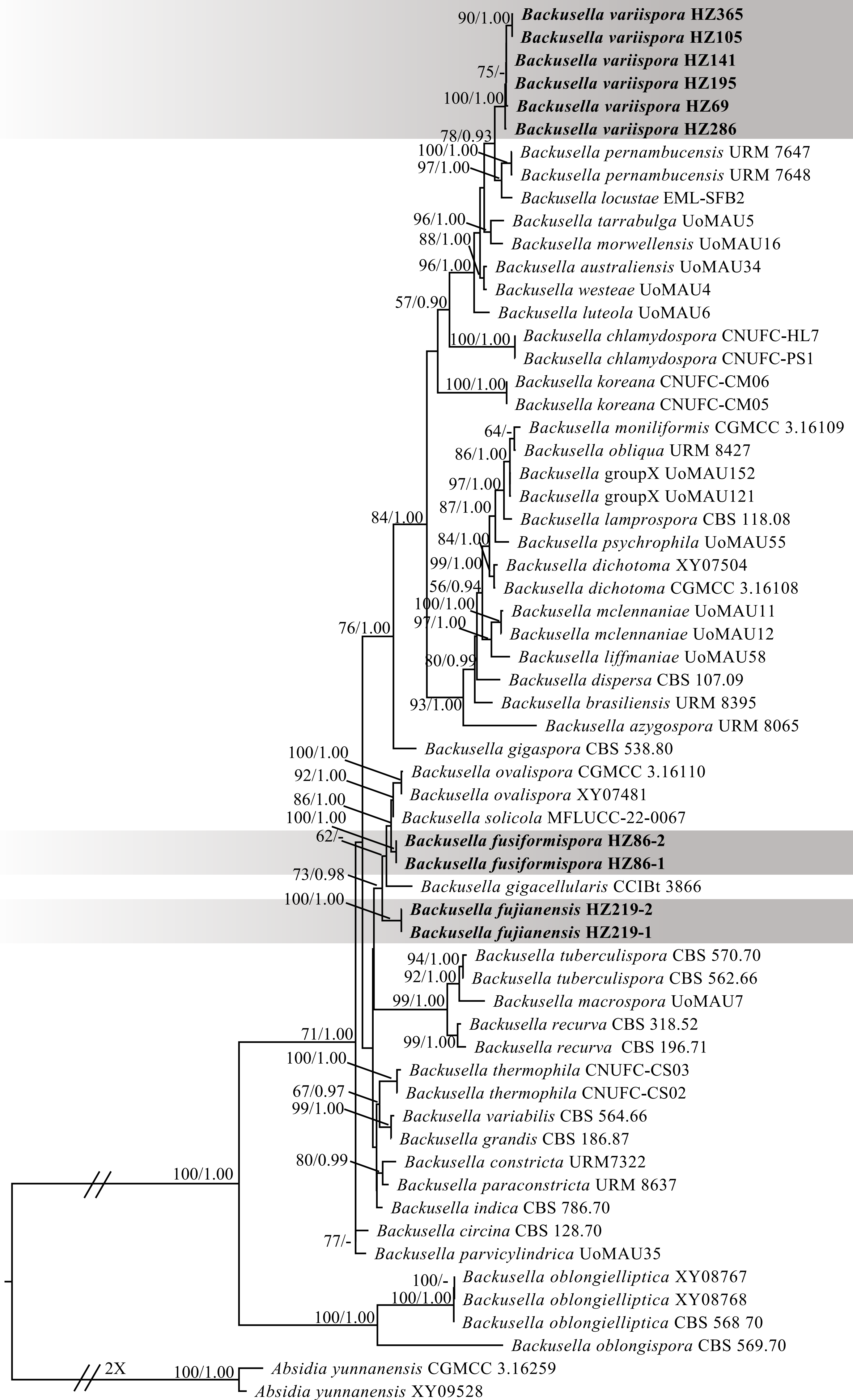

0.5

Supplement: Supplementary material 3 — The Maximum Likelihood phylogenetic tree of the genus Backusella based on ITS and LSU genetic loci [file mycokeys-109-285-s003.pdf]
